# Predicting microbe organisms using data of living micro forms of life and hybrid microbes classifier

**DOI:** 10.1371/journal.pone.0284522

**Published:** 2023-04-20

**Authors:** Ali Raza, Furqan Rustam, Hafeez Ur Rehman Siddiqui, Isabel de la Torre Diez, Imran Ashraf

**Affiliations:** 1 Department of Computer Science, Khwaja Fareed University of Engineering and Information Technology, Rahim Yar Khan, Pakistan; 2 School of Computer Science, University College Dublin, Dublin, Ireland; 3 Department of Signal Theory and Communications and Telematic Engineering, University of Valladolid, Valladolid, Spain; 4 Information and Communication Engineering, Yeungnam University, Gyeongsan, Korea; Sejong University, KOREA, REPUBLIC OF

## Abstract

Microbe organisms make up approximately 60% of the earth’s living matter and the human body is home to millions of microbe organisms. Microbes are microbial threats to health and may lead to several diseases in humans like toxoplasmosis and malaria. The microbiological toxoplasmosis disease in humans is widespread, with a seroprevalence of 3.6-84% in sub-Saharan Africa. This necessitates an automated approach for microbe organisms detection. The primary objective of this study is to predict microbe organisms in the human body. A novel hybrid microbes classifier (HMC) is proposed in this study which is based on a decision tree classifier and extra tree classifier using voting criteria. Experiments involve different machine learning and deep learning models for detecting ten different living microforms of life. Results suggest that the proposed HMC approach achieves a 98% accuracy score, 98% geometric mean score, 97% precision score, and 97% Cohen Kappa score. The proposed model outperforms employed models, as well as, existing state-of-the-art models. Moreover, the k-fold cross-validation corroborates the results as well. The research helps microbiologists identify the type of microbe organisms with high accuracy and prevents many diseases through early detection.

## Introduction

Microorganisms are the living organisms present on earth. Microorganisms are vital in medical industries to cure many diseases and maintain environmental balance [1]. The microorganism has many forms, some are beneficial, while others are harmful. The harmful microbes cause many infectious diseases and spoil other materials such as food [2]. The microbes are tiny and cannot be seen by the naked eye. A microscope is required to analyze the microorganisms. Microorganisms live everywhere, such as soil, water, and air. Scientists identified that the human body is home to millions of microorganisms. The microorganisms are of numerous types and species [3]. Each microorganism has its significant purpose. The microorganism can be detected and classified based on its shape, size, and color. The microbe’s shape can be the type of rods, spheres, and corkscrew. The microorganism has common types such as fungi [4], viruses, archaea or protists [5], algae, and bacteria [6]. The other ten most important living microforms of life are Volvox, Spirogyra, Yeast, Pithophora, Penicillium, Raizopus, Protozoa, Aspergillus sp, Ulothrix, and Diatom. These microorganisms can be identified based on microscopic data.

The microbe organisms cause many infections and diseases such as toxoplasmosis [7] and malaria [8]. According to a 2019 report, the microbiological toxoplasmosis disease is widespread in humans, with a seroprevalence of 3.6–84% in sub-Saharan Africa [9]. According to the 2020 report of the world health organization (WHO), 241 million malaria cases are found worldwide, and the number of malaria deaths is 627000 [10]. In this regard, an automatic tool for microbe organism detection would be very beneficial to save lives through the early detection of microbiological diseases.

Machine learning and deep learning have witnessed widespread use over the past decade. Artificial intelligence-based tools and techniques are widely used to process, and analyze massive amounts of medical data [11]. Artificial intelligence helps in bioinformatics for decisions making in numerous diseases using predictive analysis. Disease prediction and medical image processing [12] are the primary applications of artificial intelligence. Artificial intelligence algorithms provide the best performance on large-scale data such as the data of microorganisms [13]. With their wide deployment and superior performance, machine learning models have been adopted in disease prediction and biomedical data analytics. Researchers mostly used classical machine learning models for predicting the microorganisms in previously published studies. The prediction performance of previous studies is low using the classical machine learning models. The ensemble learning techniques were applied to enhance the prediction performance task. Keeping in view their outstanding results, this study follows a machine learning-based approach and makes the following primary contributions toward the prediction of the microbe organisms

- Microbe exploratory data analysis (MEAA) is applied to determine the dataset patterns and valuable insights for predicting the microbe organisms. The MEAA is based on the data graphs and charts representing the relations of dataset features.
- A novel hybrid microbes classifier (HMC) is proposed based on a decision tree classifier (DTC) and extra tree classifier (ETC) techniques for predicting microbe organisms. The final prediction is made using the voting criterion. Experiments involve multi-class classification with ten classes including Aspergillus sp, Diatom, Penicillium, Pithophora, Protozoa, Raizopus, Spirogyra, Ulothrix, Volvox, and Yeast.
- Ten machine learning and deep learning-based models are applied in comparison to the proposed approach for predicting microbe organisms. The multi-layer perceptron classifier (MLP), DTC, random forest classifier (RFC), logistic regression (LR), k-nearest neighbors (KNN), gradient boosting classifier (GBC), ETC, and support vector machines (SVM) are employed in this regard. Also, long short-term memory (LSTM) and gated recurrent unit (GRU) is used as the deep learning models. The performance is analyzed with respect to the accuracy, precision, recall, F1 score, and k-fold cross-validation

The remainder of this study is organized as follows. Section 2 is based on the related literature analysis. The methodology and proposed approach are discussed in Section 3. Experimental results and discussions are given in Section 4. Finally, the study is concluded in Section 5.

## Related work

The identification of microbial contaminants in the pharmaceutical industry using a deep learning-based approach is studied in [14]. The Raman spectroscopy dataset is utilized to build the deep learning model. The dataset target microbial contaminants are gram-positive bacteria, gram-negative bacteria, and fungi. The convolution neural network (CNN) is used for experiments which achieve a 95% accuracy score for microbial contaminants prediction. The prediction of personalized antibiograms in microbiology using machine learning is carried out in [15]. The electronic health record data of 8342 infections and 15806 uncomplicated urinary tract infections is utilized for the model building. The gradient boosted tree (GBT) shows outstanding results among the employed machine learning models. The personalized antibiograms performance coverage rate is 90% using the proposed technique.

The generation and classification of microbial colonies images using deep learning-based models is studied in [16]. The synthetic microbial colonies dataset of Petri dishes [17] is utilized. The multi-class data of five different microbial species are utilized for classification. The R-CNN model is employed for generating and detecting microbial colonies. The proposed approach achieved a mean squared error score of 4.49 and a mean average precision accuracy score of 0.520.

The study [18] performs the detection of candida albicans fluconazole resistance using a machine learning approach. The combined dataset based on matrix-assisted laser ionization (MALI), time-of-flight (TOF), and mass spectrometry (MS) is utilized for building machine learning models. The authors leverage the linear discriminant analysis (LDA) for the detection of candida albicans which yields an 85% accuracy. Similarly, [19] proposed the detection of carbapenem-resistant Klebsiella pneumoniae in microbiology using a supervised machine learning approach. The MALDI-TOF MS data is utilized in this research. The study proposes a modified random forest (RF) technique that achieves an accuracy score of 97% for the detection task.

The prediction of methicillin-resistant Staphylococcus aureus using machine learning methods is studied in [20]. The MALDI-TOF MS spectrum data is utilized with the SVM model. Results show an accuracy of 86% using the SVM. The authors study the classification of group B Streptococcus serotypes in [21]. The MALDI-TOF MS data is utilized with SVM and RF models. Results suggest that the RF model outperforms with an accuracy score of 87%.

Skin syndrome detection based on deep neural networks is presented in this study [22]. The deep learning-based techniques MobileNet and long short-term memory (LSTM) are utilized to classify skin disease in real time. The proposed model achieved 85% accuracy on the HAM10000 dataset. However, it can be further improved by fine-tuning different parameters. The automatic detection of Alzheimer’s disease using the fusion-based approach with a heterogeneous ensemble classifier is proposed in [23]. The proposed framework is utilized to predict Alzheimer’s disease based on multimodal time-series data. The dataset is based on 1371 subjects from the Alzheimer’s disease neuroimaging initiative (ADNI). Experimental results show that the proposed model achieves superior results in comparison with the state-of-the-art technique for Alzheimer’s prediction.

The related literature in the context of predicting microbe organisms is examined in this section. The related research proposed approach, dataset, performance score, and the main aim of the research are analyzed. The past applied state-of-the-art approaches are comparatively analyzed in Table 1.

**Table 1:** The analysis of related literature in the context of predicting the microbe organisms.

Ref.	Year	Approach	Dataset	Accuracy %	Aim
[14]	2020	CNN	Raman spectroscopy	95	Identifying microbial contaminants in the pharmaceutical industry using a deep learning-based approach.
[15]	2022	GBT	Electronic health record data	90	The prediction of personalized antibiograms in microbiology using machine learning was proposed.
[16]	2022	R-CNN	Synthetic microbial colonies	52	The generation and classification of microbial colonies images using deep learning-based models were proposed.
[18]	2020	LDA	MALI- TOF- MS	85	The detection of candida albicans fluconazole resistance in microbiology using machine learning was proposed.
[19]	2020	RF	MALI- TOF- MS	97	This study proposed the detection of carbapenem-resistant Klebsiella pneumoniae in microbiology using a supervised machine learning approach.
[20]	2018	SVM	MALDI-TOF MS spectrum	86	The prediction of methicillin-resistant Staphylococcus aureus using machine learning methods was proposed.
[21]	2019	RF	MALDI-TOF MS	87	The classification of group B Streptococcus serotypes using machine learning was proposed.

## Study methodology

**Fig 1:**
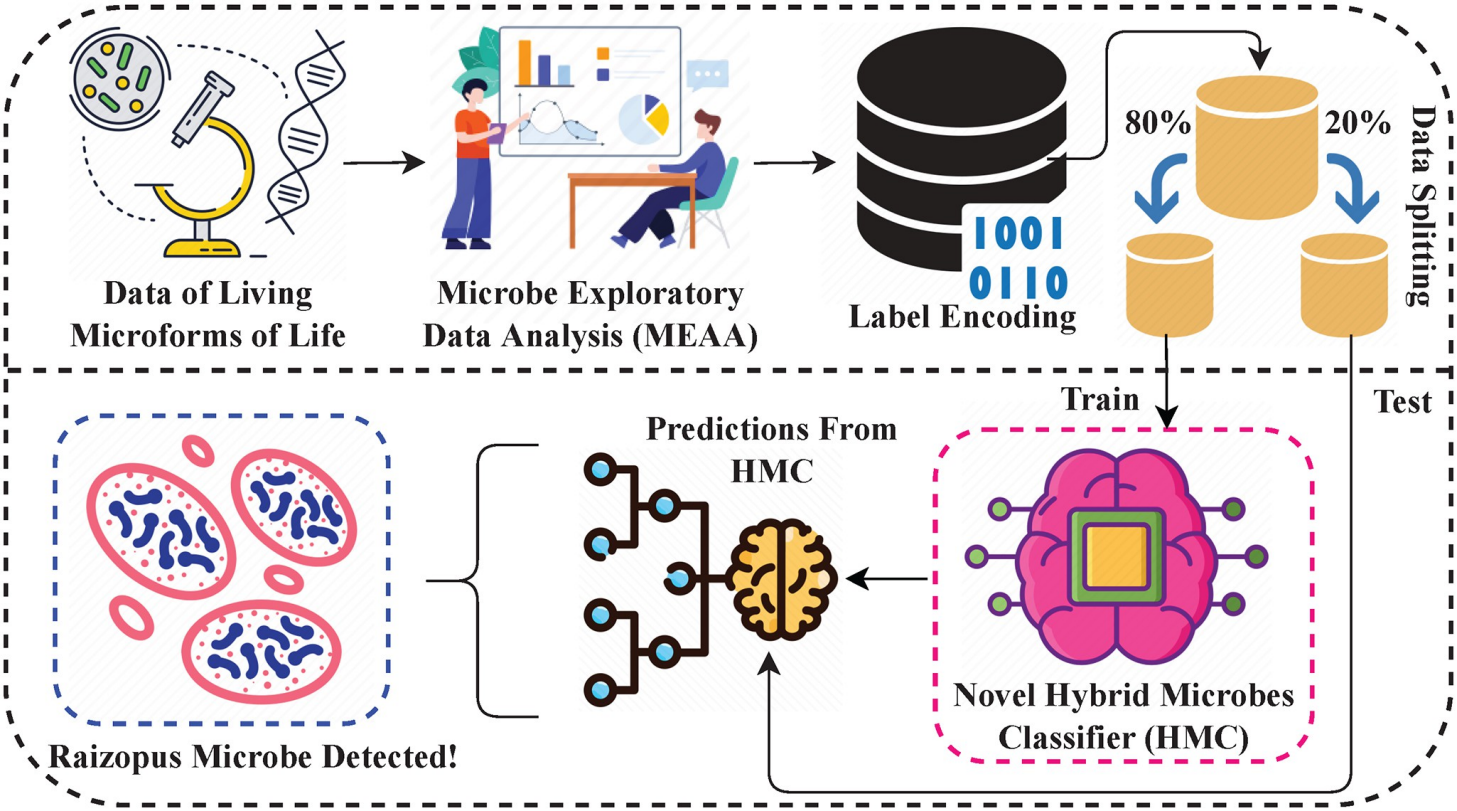
The architecture of the proposed approach for predicting microbe organisms. It involves data collection, exploratory data analysis, model training and testing.

The methodological analysis of the proposed approach for predicting the microbe organisms in microbiology is visualized in Fig 1. The data of different living microforms of life is utilized for conducting the research experiments. The MEDA is applied to obtain critical insights and patterns in predicting the microbes. The target class in data is encoded to numeric form to transform the labels into machine readable form. The preprocessed data is split into train and test portions with a ratio of 0.8 to 0.2. The novel proposed HMC approach is trained with 80% of data and evaluated using 20% of unseen test data. The proposed HMC approach is fully hyper parameterized to obtain the best results.

### Microbe organisms data

The research utilizes the data of different living microforms of life that is publicly available at Kaggle [24] and used in a DPhi challenge [25]. The data contains ten different living microforms of life, which are Volvox, Spirogyra, Yeast, Pithophora, Penicillium, Raizopus, Protozoa, Aspergillus sp, Ulothrix, and Diatom. The description of the different features, types, and counts are given in Table 2. The utilized dataset features are based on the 21368 microscopic object images of different living microforms of life. The dataset is based on the 25 microscopic object features which are used to predict microbe organisms in our research study.

**Table 2:** Description of dataset features.

Sr no.	Feature	Non-null count	Data type	Description
1	Solidity	21368	float64	The solidity is the ratio of the area of an object to the area of a convex hull of the object.
2	Eccentricity	21368	float64	The eccentricity is the ratio of the length of the major to the minor axis of an object.
3	EquivDiameter	21368	float64	The diameter of a circle with the same area as the region.
4	Extrema	21368	float64	The extrema are the points in the region.
5	FilledArea	21368	float64	The number of pixels in the filled image returned as a scalar.
6	Extent	21368	float64	The ratio of the pixel area of a region with respect to the bounding box area of an object.
7	Orientation	21368	float64	The overall direction of the shape. The value ranges from -90 degrees to 90 degrees.
8	EulerNumber	21368	float64	The number of objects in the region minus the number of holes in those objects.
9	BoundingBox1	21368	float64	Position and size of the smallest box (rectangle) which bounds the object.
10	BoundingBox2	21368	float64	
11	BoundingBox3	21368	float64	
12	BoundingBox4	21368	float64	
13	ConvexHull1	21368	float64	Smallest convex shape/polygon that contains the object.
14	ConvexHull2	21368	float64	
15	ConvexHull3	21368	float64	
16	ConvexHull4	21368	float64	
17	MajorAxisLength	21368	float64	The major axis is the endpoints of the longest line that can be drawn through the object. The length (in pixels) of the major axis is the largest dimension of the object.
18	MinorAxisLength	21368	float64	The axis perpendicular to the major axis is called the minor axis. The minor axis’s length (in pixels) is the smallest line connecting a pair of points on the contour.
19	Perimeter	21368	float64	The number of pixels around the border of the region.
20	ConvexArea	21368	float64	Centre of mass of the region. It is a measure of an object’s location in the image.
21	Centroid1	21368	float64	The centre point of the object.
22	Centroid2	21368	float64	
23	Area	21368	float64	The total number of pixels in a region/shape.
24	raddi	21368	float64	The radius of the object.
25	microorganisms	21368	object	The microorganism target class to where they belong.

### Microbe exploratory data analysis

MEDA is applied to the research dataset to determine patterns and valuable insights in predicting microbe organisms. The graph and chart-based MEDA are performed, representing the relations of dataset features.

**Fig 2:**
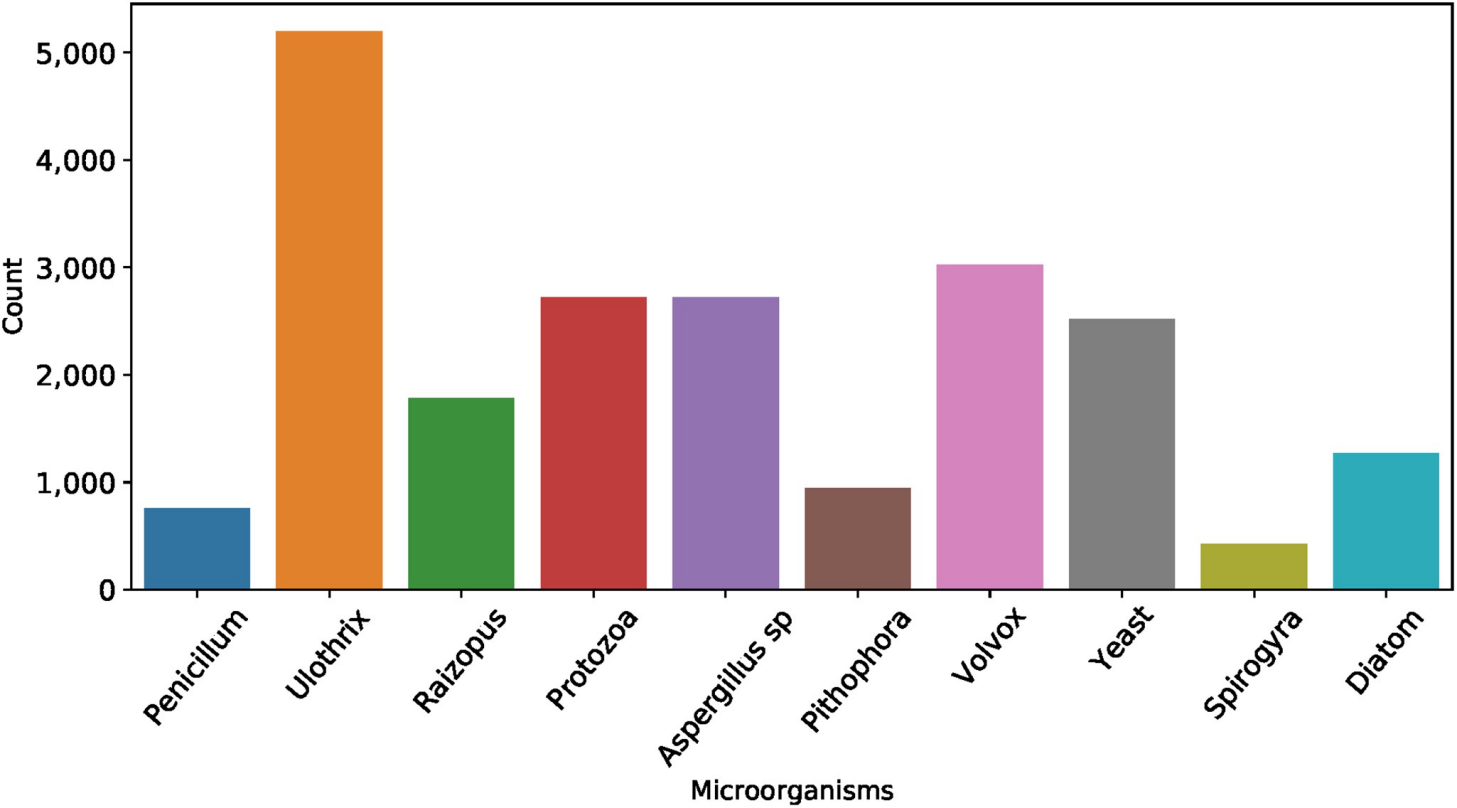
The bar chart-based frequency analysis of each microorganism target label showing the number of samples in each class.

The bar chart-based microorganisms target label frequency analysis is performed in Fig 2. The frequency for each label is represented in the chart’s *y*-axis. The analysis demonstrates that the target label Ulothrix contains 5194, Volvox contains 3024, Protozoa contains 2721, Aspergillus sp contains 2721, Yeast contains 2520, Raizopus contains 1786, Diatom contains 1273, Pithophora contains 945, Penicillum contains 756, and Spirogyra contains 428 instances. This analysis shows that Ulothrix class contains a high number of instances, and Spirogyra contains the lowest number of instances.

**Fig 3:**
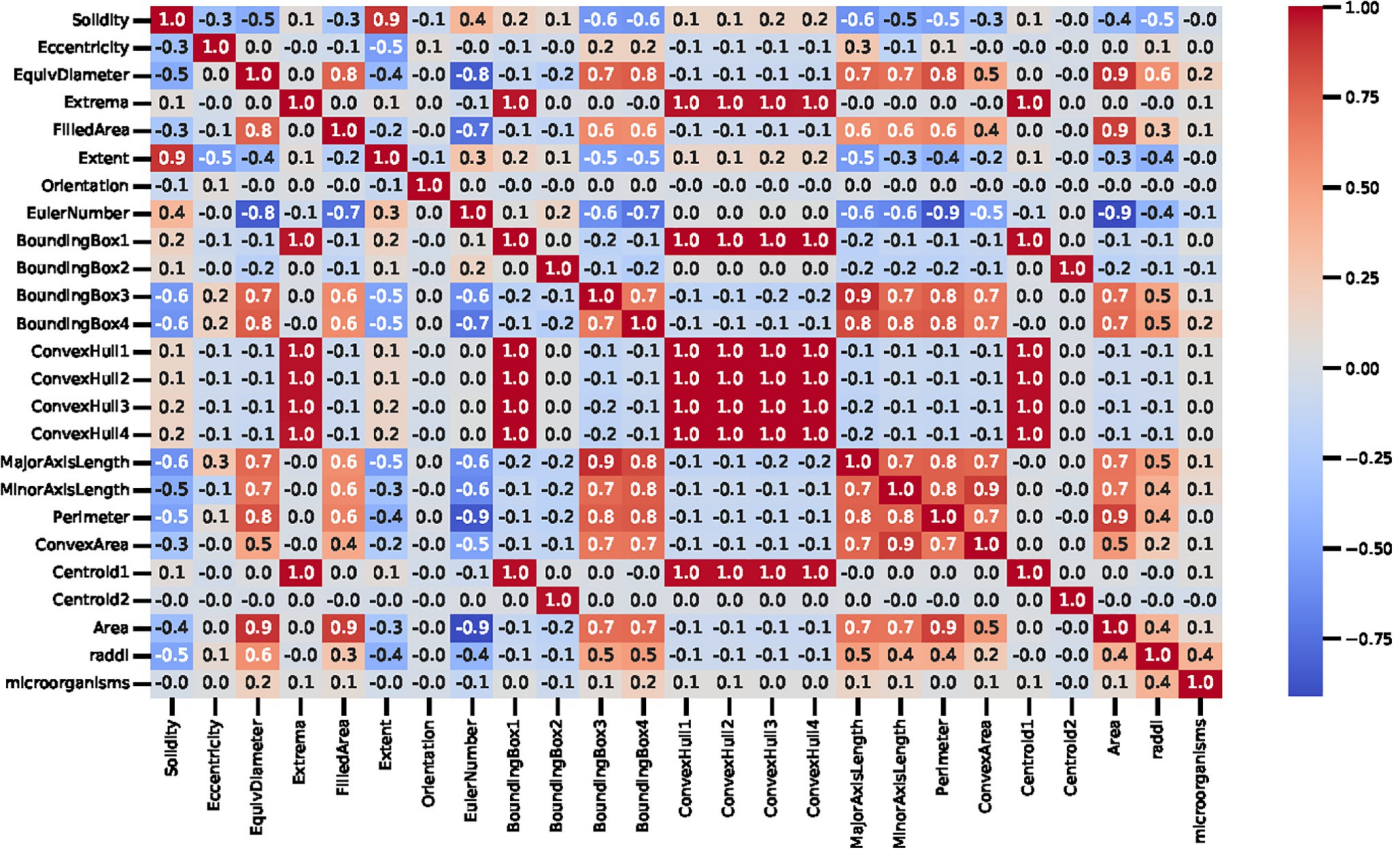
The correlation analysis of employed dataset features indicating the importance of features regarding the target class.

The statistical correlation analysis is visualized in Fig 3. The correlation is utilized to determine the linear relationship between two dataset features and analyze their association. This explains how features are related to each other. The analysis demonstrates that the features Extrema, BoundingBox, ConvexHull, and centroid have high correlation values. The features MajorAxisLength, MinorAxisLength, Perimeter, and ConvexArea also have good correlations association. The features Solidity, Extent, and EulerNumber have negative correlation values.

**Fig 4:**
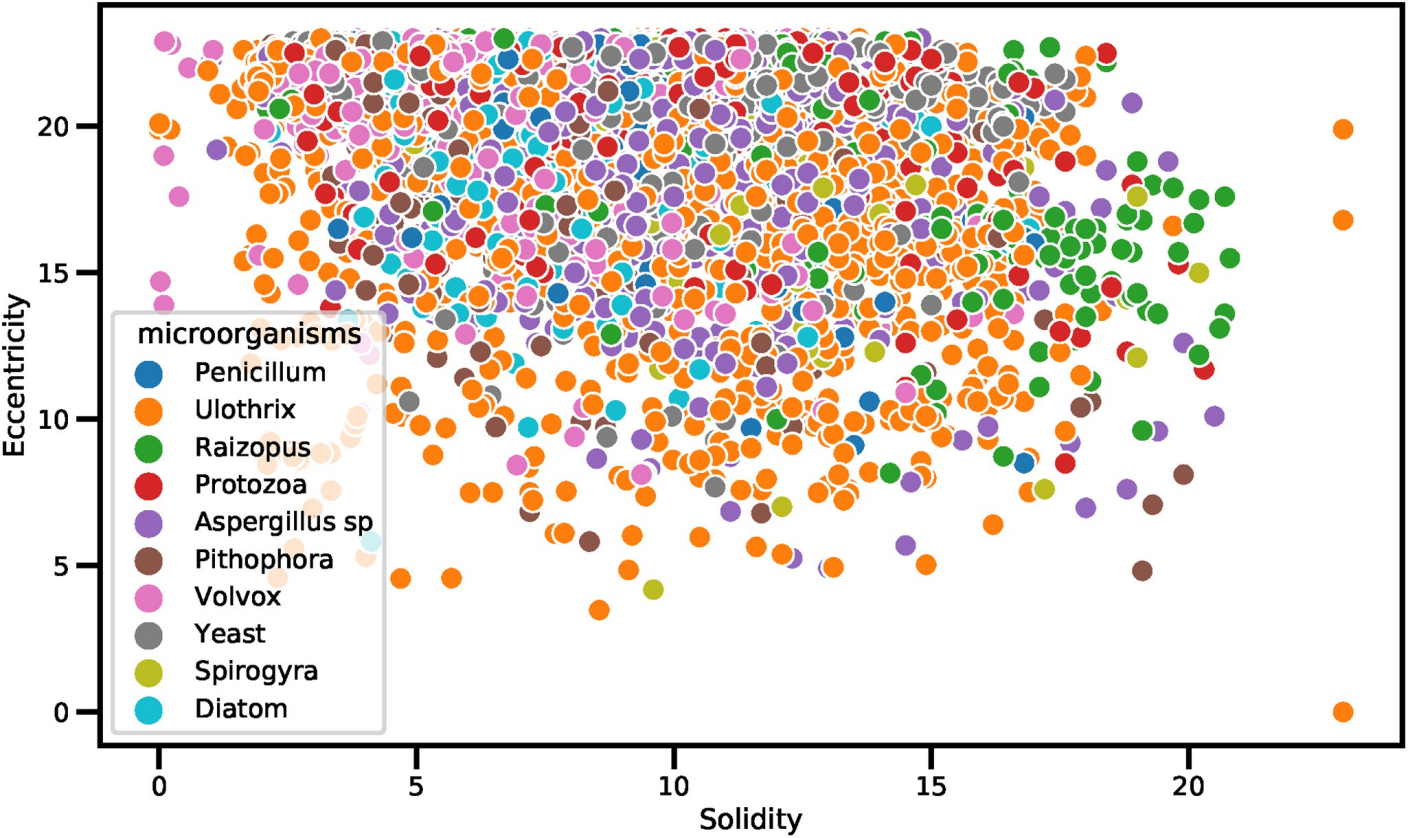
The scatter plot showing the distribution of features regarding Solidity and Eccentricity along with the target class.

**Fig 5:**
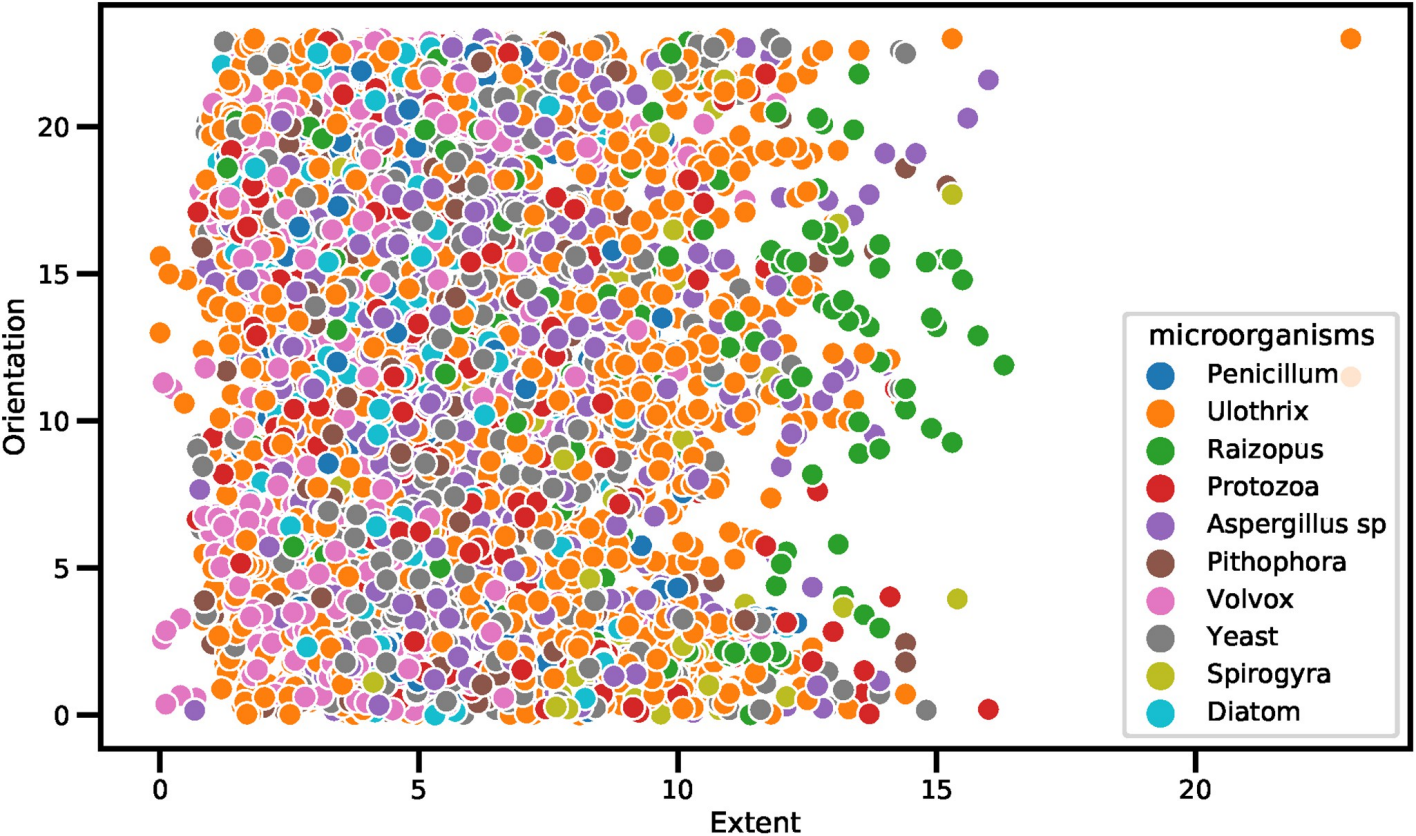
The scatter showing the distribution of features regarding Extent and Orientation along with the target class.

The scatter plot-based analysis of different data features is shown in Figs 4 and 5. The scatter plot is primarily utilized to determine the relationships between two dataset features. The dot values in the scatter plot represent the patterns involved in the prediction process. The purpose of the scatter plot is to observe the relation when the values of features change. The scatter plot analysis of features Solidity and Eccentricity along with the target class is visualized in Fig 4. The analysis demonstrates that the microorganisms have the Solidity and Eccentricity feature values in the range of 5 to 30. The analysis shows that the Raizopus microbe is identified when the Solidity values are above 15 and less than 20. All other microbes are identified when the Solidity values are less than 18 and Eccentricity values are above 5. There is a high chance of microorganism detection when the Eccentricity values are above 15 and the Solidity values are above 3.

The scatter plot analysis of features Extent and Orientation along with the target class is visualized in Fig 5. The analysis demonstrates that the microorganisms have Extent feature values in the range of 0 to 20 and Orientation feature values in the range of 0 to 30. The analysis shows that the microorganisms are identified when the Extent values are between 0 and 15. The high chances of Raizopus microbe detection when the Extent values are above 10.

### Label encoding and data splitting

We have transformed the dataset target class labels into the machine-readable numeric form using the label encoding technique. The label encoder module from scikit-learn is utilized for the encoding process. The module encodes the target labels with a value between 0 and the total number of classes. Data splitting is a crucial part of machine learning which is applied to split the data into training and testing sets. We split the microbe dataset into 80–20 train-test splits.

### Proposed hybrid classifier

**Fig 6:**
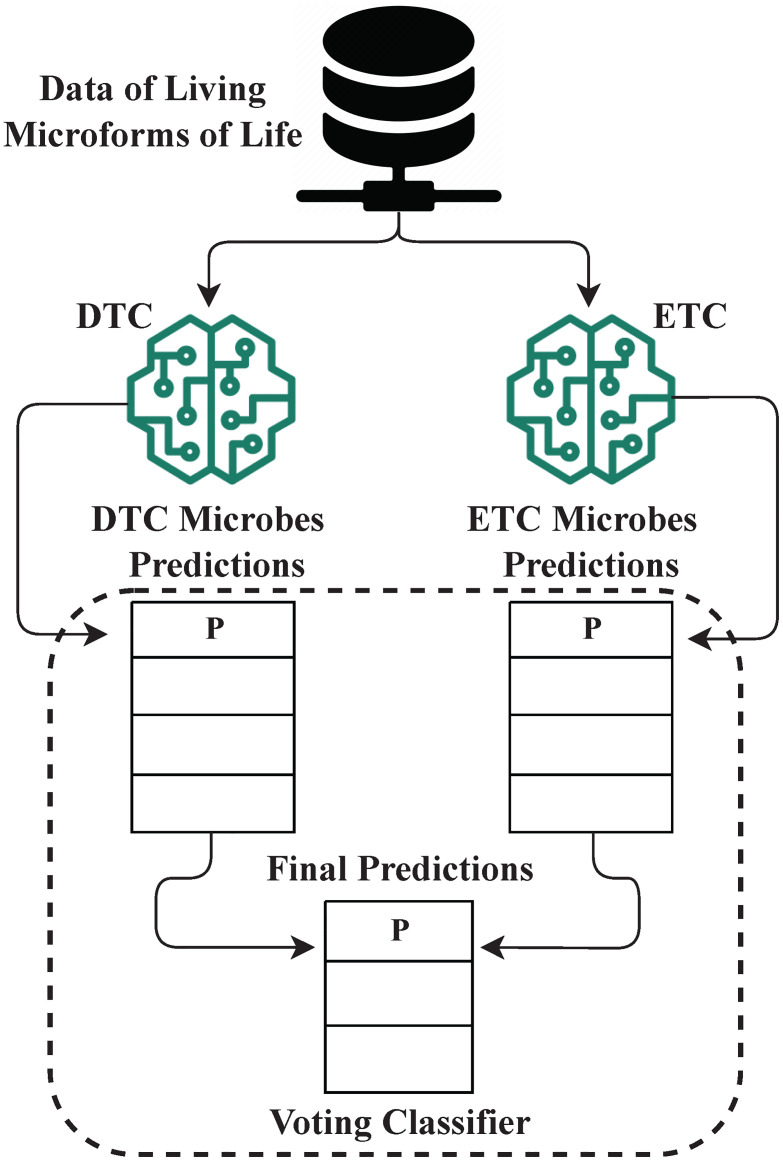
The architecture of the proposed HMC approach showing the voting process for the hybrid classifier.

A novel HMC is proposed based on a hybrid of DTC and ETC for predicting microbe organisms. The architecture of the proposed HMC approach is shown in Fig 6. The data of different living microforms of life is input to both DTC, and ETC approaches. The DTC and ETC are combined to predict the microbe organisms. The class with the majority of votes from individual predictions is taken to make the final prediction using voting. The final predictions are obtained by using ‘hard’ voting.

The proposed hybrid classifier is based on the combination of multiple supervised classifiers. The key objective of the proposed ensemble method is to reduce variance and bias thus enhancing the prediction performance. The ensemble hybrid methods are proven to show better performance where the dataset has a higher number of features. The predictions of each classifier are passed to the voting classifier to predict the output class based on the majority voting. The prediction performance is improved by resolving the error of each classifier during voting.

### Employed machine learning models

The applied machine learning and deep learning models for predicting microbe organisms in microbiology are analyzed in this section.

The DTC is a supervised machine learning model commonly used to solve classification problems [26]. The DTC follows the tree structure to make a decision on data samples. The leaf nodes in the tree contain the target class labels, the tree branches represent the decision rules, and the internal nodes contain the data attributes. The Gini index is mainly utilized in DTC to select the best data attributes during tree constructions as expressed in Eq 1, where *p* represents the probability of data attributes.

$$\begin{array}{c} Gini\;index=1-\sum_{j}\;P_{j}^{2} \end{array}$$ (1)

RFC is an ensemble learning model which utilizes decision trees [27]. The RFC model works similarly to the DTC model. In the RFC model, multiple decision trees are created for prediction tasks instead of creating a single tree. The prediction outcomes from multiples tree are taken to make the final prediction. RFC helps to improve the prediction accuracy and control model over-fitting.

ETC is also an ensemble learning method widely used for the classification task [28]. The bagged decision trees are constructed in the ETC model for prediction. ETC is similar to the RFC model. The only difference is the tree-based forest construction in the ETC model. The predictions from multiple de-correlated decision trees are aggregated to make the final prediction.

GBC is an ensemble Learning model [29]. The GBC model combines multiple weak classifiers into a robust classifier to obtain high accuracy. During training, each weak classifier improves accuracy and reduces errors. The gradient boosting is based on the decision trees.

KNN is a non-parametric learning classifier mainly used for classification and regression problems [30]. The KNN model makes the groups of data have similar properties. The Euclidean distance metric is utilized to find the similarity between data points. For each data point, the Euclidean distance values are determined by the data points near it.

LR is another widely used supervised method primarily used to solve classification problems [31]. LR model determines the relationship between the independent and dependent variables. LR is a statistical method that utilizes a logistic sigmoid function for classification tasks. The probabilistic values lie between zero and one for using the logistic sigmoid function. Eq 2, represents the prediction process by the LR model.

$$\begin{array}{c} y=\frac{e^{b_{0}+b_{1}*z}}{1+(e^{b_{0}+b_{1}*z})} \end{array}$$ (2)

where *y* is the predicted class, *b*_0_ is the bias term, and *b*_1_ is the coefficient for input *x*.

SVM is a supervised method that utilizes the support vectors to classify the data points [32]. The primary motive of the SVM model is to determine the best-fit decision boundary. The best-fit decision boundary classifies the *n*-dimensional feature space data into the target label. The best-fit decision boundary is also known as the hyperplane [33]. The error is minimized by the iterative process of finding the best-fit decision boundary. SVM selects the extreme support vectors to create the hyperplane. The best-fit hyperplane is represented in Eq 3.

$$\begin{array}{c} \vec{w}.\vec{x}+b=0 \end{array}$$ (3)

where *w* represents the weight matrix, *x* represents the input features and *b* indicates the biased values.

MLP is a feedforward artificial neural network-based supervised learning model [34]. The artificial neural network uses many representation layers to process the data. The model layers contain neuron units in the network. The layers have the graph representation between the input and output layers. The backpropagation technique [35] is utilized in the MLP model to train the network.

LSTM model is a recurrent neural network known best for learning long-term sequences [36]. The primary motive behind the LSTM model is to remember the long sequences for a long period. The LSTM model contains three gates for processing: input gate, output gate, and forget gate. The LSTM model has a high number of training parameters that use high memory.

GRU model is a recurrent neural network [37]. It contains two gates: the update gate and the reset gate which are utilized for its working mechanism. The GRU model has less complexity than the LSTM model due to a smaller number of gates. The GRU model uses fewer training parameters that use less memory and execute faster. The GRU and LSTM model benefit from overcoming the vanishing gradient problem.

The hyperparameter tuning and optimization techniques [38] is based on the iterative process of training and evaluation of learning models. In the iterative tuning process, the parameters on which the learning model gives the best performance accuracy scores are considered the best-fit hyperparameters. The best-fit hyperparameters result in higher accuracy scores for predicting the microbe organisms in this study. The final selected hyperparameters for learning models are given in Table 3.

**Table 3:** The hyperparameters of employed learning techniques.

Technique	Hyperparameters
MLP	Hidden_layer_sizes = 80, Max_iter = 100, Solver = adam, Activation = relu, Alpha = 0.0001, Learning_rate_init = 0.001, Learning_rate = constant, Tol = 1e-4, Epsilon = 1e-8, Momentum = 0.9, Max_fun = 15000.
DTC	Criterion = entropy, Splitter = best, Max_depth = 20, Ccp_alpha = 0.0, Random_state = 0, Min_samples_split = 2, Min_samples_leaf = 1, Max_features = None.
RFC	N_estimators = 20, Max_depth = 20, Criterion = gini, Max_features = 1.0, Bootstrap = True, Ccp_alpha = 0.0, Random_state = 5.
LR	Random_state = 10, Solver = lbfgs, Max_iter = 100, Multi_class = auto, C = 1.0.
KNN	N_neighbors = 5, Weights = uniform, Leaf_size = 30, P = 2, Metric = minkowski.
GBC	N_estimators = 20, Max_depth = 20, Learning_rate = 0.01, Loss = log_loss, Criterion = friedman_mse.
ETC	N_estimators = 20, Random_state = 0, Max_depth = 20 Criterion = gini, Max_features = sqrt.
SVM	Random_state = 50, Max_iter = 100, Penalty = l2, Loss = squared_hinge, Tol = 1e-4, C = 1.0, Multi_class = ovr.
LSTM	Loss = categorical_crossentropy, Optimizer = adam, Metrics = accuracy, Activation = softmax.
GRU	Loss = categorical_crossentropy, Optimizer = adam, Metrics = accuracy, Activation = softmax.

## Results and discussions

Results and discussions are presented in this section. The results of all the machine learning and deep learning models are compared. The performance evaluation is based on accuracy, error rate, precision, recall, F1, cohen kappa, and the geometric mean score.

### Experimental setup

The Python 3.0 programming tool [39] is utilized to conduct all experiments. The modules Keras version 2.8.0 and TensorFlow version 2.8.2 are used for building deep learning models. Machine learning models are built using the Scikit-learn module version 1.0.2. The platform with 13GB RAM and a 2.20GHz CPU is used to complete the experiments.

### Results of machine learning and deep learning models

Experimental results of all the models are given in Table 4. Results indicate that the proposed approach obtains the best results with 98% accuracy and geometric mean,97% precision and Cohen Kappa, and 96% recall and F1 scores. Regarding the training time, propose approach takes 1.386 seconds which is higher than only KNN, ETC, and DTC which take 0.028, 0.522, and 1.242 seconds, respectively.

**Table 4:** Performance analysis of employed machine and deep learning techniques with the proposed technique.

Technique	Training time (sec)	Accuracy	Error rate	Precision	Recall	F1 score	Cohen Kappa	Geometric mean
MLP	34.665	62	0.381	61	57	58	55	73
DTC	1.242	97	0.025	97	95	96	97	97
RFC	1.437	97	0.026	98	95	96	96	97
LR	4.349	44	0.558	36	33	32	33	55
KNN	0.028	88	0.115	88	85	86	86	91
GBC	88.761	96	0.032	98	94	96	96	96
ETC	0.522	97	0.025	98	95	96	97	97
SVM	3.747	41	0.592	39	38	36	31	59
LSTM	72.297	30	0.697	65	30	41	49	44
GRU	83.966	34	0.658	74	34	47	64	50
**Proposed**	**1.368**	**98**	**0.024**	**97**	**96**	**96**	**97**	**98**

**Fig 7:**
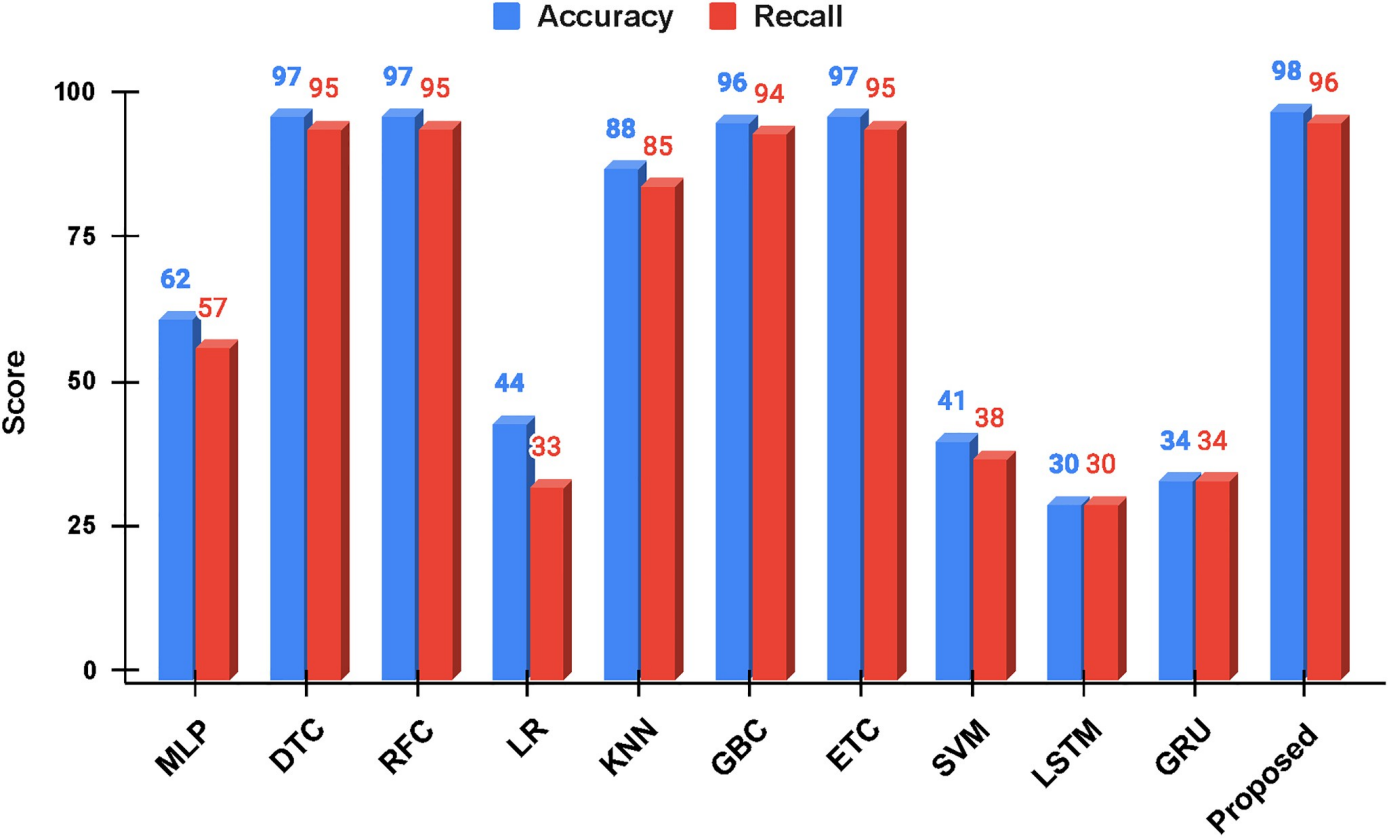
Comparative analysis of employed machine learning and deep learning models in terms of accuracy and recall.

The second best accuracy is obtained jointly by the DTC, RFC and ETC which obtain 97% accuracy, as shown in Fig 7. Machine learning models tend to perform better on average, except for SVM and LR which obtains 41% and 44% accuracy, respectively. Deep learning models show poor performance and obtain the lowest accuracy scores of 30% and 34% for LSTM and GRU models. Due to the smaller dataset, the models can not get a good fit and show poor results.

**Fig 8:**
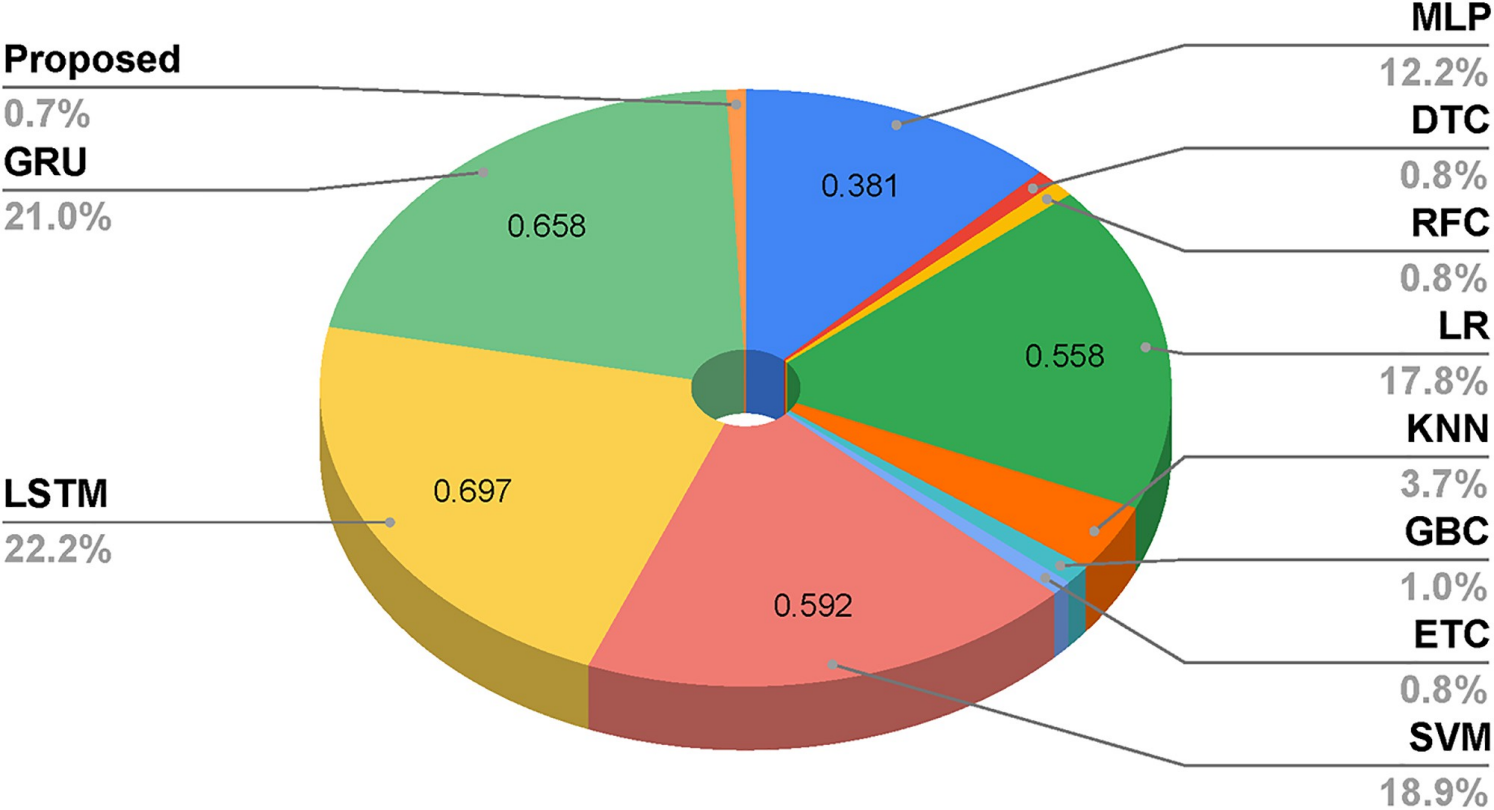
Comparative analysis of employed machine learning and deep learning models in terms of prediction error rate.

The pie chart-based error rate comparative analysis of employed learning techniques is visualized in Fig 8. The analysis demonstrates that the proposed approach has the minimum error rate indicating high-performance accuracy scores for the microbe organism predictions. Based on this analysis, the proposed approach has a 0.7% error rate. The high error rate of 22% is achieved by the LSTM model, which indicates the low accuracy scores. The analysis shows that DTC and RFC have the same error rate of 0.8%, indicating maximum accuracy scores.

The classification report based on individual categories is given in Table 5. The analysis demonstrates that the organism’s categories Penicillum and Raizopus achieved a 100% score for all performance metrics. The categories Protozoa and Raizopus achieved 100% scores for recall and F1 score measures, respectively. The average performance metrics scores for all are between 96% to 97%. This analysis validates the proposed model results and demonstrates the high accuracy scores for the microbe’s organism’s predictions.

**Table 5:** Individual class-vise report of the proposed approach.

Category	Precision	Recall	F1-score	Support
Aspergillus sp	0.94	0.98	0.96	523
Diatom	0.93	0.97	0.95	266
Penicillum	1.00	1.00	1.00	146
Pithophora	0.91	0.87	0.89	178
Protozoa	0.99	1.00	1.00	546
Raizopus	1.00	1.00	1.00	350
Spirogyra	0.95	0.80	0.87	71
Ulothrix	0.97	0.97	0.97	1041
Volvox	0.99	1.00	0.99	636
Yeast	1.00	0.97	0.99	517
**Average**	**0.97**	**0.96**	**0.96**	**4274**
**Accuracy**	**0.98**			

### Results of k-fold cross-validation

The k-fold cross-validation results of employed learning techniques are given in Table 6. The 10-fold cross-validation results demonstrate that the proposed approach achieves a high accuracy score of 98%. The standard deviation score of the proposed approach is ±0.0033, which is the minimum compared to other techniques. The lowest accuracy score is archived by the SVM technique, which is 24% for 10-fold cross-validation. This analysis validates that the proposed model can provide generalized results for predicting microbe organisms.

**Table 6:** K-fold cross-validation results of employed models.

Techniques	K-Fold	Accuracy (%)	Standard Deviation
MLP	10	61	±0.0107
DTC	10	97	±0.0038
RFC	10	97	±0.0029
LR	10	43	±0.0095
KNN	10	89	±0.0081
GBC	10	97	±0.0041
ETC	10	97	±0.0037
SVM	10	24	±0.0729
LSTM	10	81	±0.1224
GRU	10	86	±0.1119
**Proposed**	**10**	**98**	**±0.0033**

### Comparison with state-of-the-art approaches

The comparative performance analysis of other state-of-the-art studies is given in Table 7. The state-of-the-art studies from 2019 to 2022 are considered. These studies employ different models line RF, logit boost, KNN, and GRU. For a fair comparison, the models are implemented on the dataset used in this study. Accuracy, recall, and geometric mean scores are utilized for comparison. The analysis demonstrates that the proposed approach outperforms the state-of-the-art studies with high accuracy for predicting microbe organisms.

**Table 7:** Performance analysis of the proposed approach with state-of-the-art studies.

Ref.	Year	Technique	Accuracy (%)	Recall (%)	GM (%)
[40]	2019	Random Forest	97	95	97
[41]	2019	Random Forest	97	95	97
[42]	2019	logit boost	61	58	74
[43]	2021	k-nearest neighbors	88	85	95
[44]	2022	Gated recurrent units	34	34	50
[45]	2021	Random Forest	97	95	97
**Proposed**	**2022**	**HMC**	**98**	**96**	**98**

### Discussion

The prediction of the microbe organisms using the data of different living microforms of life is presented in this study. An ensemble method based on a hybrid of DTC and ETC techniques is used for the prediction task. Experiments are performed using many machine learning and deep learning models for performance comparisons like DTC, RFC, LR, KNN, GBC, ETC SVM, MLP, LSTM, and GRU. These models are optimized regarding different hyperparameters to obtain the best results. For performance analysis, Cohen Kappa and geometric mean are used in addition to error rate, accuracy, recall, precision, and F1 score. Moreover, training time is also used to estimate the computational complexity of models. Results reveal that DTC, RFC, and ETC obtain the best results among machine learning models with moderate training time. On the other hand, deep learning models show poor performance and have a higher training time. The proposed approach obtains the best performance compared to both machine learning and deep learning models with 98% accuracy and geometric mean each. In addition, its error rate of 0.024 is also the lowest among all models. K-fold cross-validation proves the robustness of the proposed approach. Similarly, performance comparison with existing state-of-the-art studies shows that the results from the proposed approach are superior. The research study helps microbiologists for the identification of different types of microbe organisms with high accuracy.

## Conclusions

The human body contains millions of microbe organisms that carry out both positive and negative activities. Microbe organisms can cause different infections and diseases and their prediction can be vital for the early detection of diseases. This study proposes an automatic approach for the prediction of ten types of microbe organisms like Aspergillus sp, Diatom, Penicillum, Pithophora, Protozoa, Raizopus, Spirogyra, Ulothrix, Volvox, and Yeast. The proposed hybrid approach, comprising DTC and ETC, shows better accuracy than employed machine learning and deep learning models and obtains a 98% accuracy. Similarly, the geometric mean, recall, precision, and F1 scores are the best among all the models and it obtains the lowest error of 0.024. K-fold cross-validation and performance comparison with state-of-the-art methods further validate its superior performance. Owing to the poor performance of deep learning models, we intend to incorporate a large dataset in the future. Similarly, using transfer learning and multi-class data balancing is also intended.

## Supporting information

S1 Dataset (ZIP)
